# Severe acne induced by clascoterone: A case report

**DOI:** 10.1016/j.jdcr.2026.02.019

**Published:** 2026-02-16

**Authors:** Alejandra Logreira Castillo, Maria Camila Amaya Muñoz, Silvia Juliana Castillo Garavito, Ricardo Flaminio Rojas Lopez

**Affiliations:** a Universidad Autónoma de Bucaramanga, Floridablanca, Colombia; b Universidad del Rosario, Bogotá, Colombia

**Keywords:** acne vulgaris, adverse drug reaction, antiandrogens, clascoterone, medical dermatology, paradoxical reaction, topical therapy

## Introduction

Acne vulgaris is a multifactorial inflammatory disease involving sebaceous hypersecretion, follicular hyperkeratinization, inflammation, and *Cutibacterium acnes* colonization.^1^ Androgens play a key role in its pathogenesis by stimulating sebum production and proinflammatory cytokine release, making androgen inhibition an important therapeutic target.^1^^,^^2^ Systemic antiandrogens, such as combined oral contraceptives and spironolactone, have proven effective, although adverse effects limit their use, particularly in men.^3^ In 2020, the US Food and Drug Administration approved topical clascoterone 1%, a novel androgen receptor antagonist for patients ≥12 years, offering local activity with reduced systemic risk.^3^^,^^4^

Adverse reactions are usually mild and localized, including pruritus, erythema, dryness, and desquamation.^2^^,^^3^^,^^5^ Nevertheless, reports remain scarce, emphasizing the importance of documenting unexpected events.

## Case report

We present the case of a 23-year-old woman with a history of urolithiasis, menarche at 12 years of age, G0P0A0C0, and irregular menstrual cycles, who discontinued combined oral contraceptives (levonorgestrel plus ethinylestradiol) because of abnormal uterine bleeding. She had no history of hyperandrogenism, polycystic ovarian syndrome, infertility, or severe acne.

**Fig 1:**
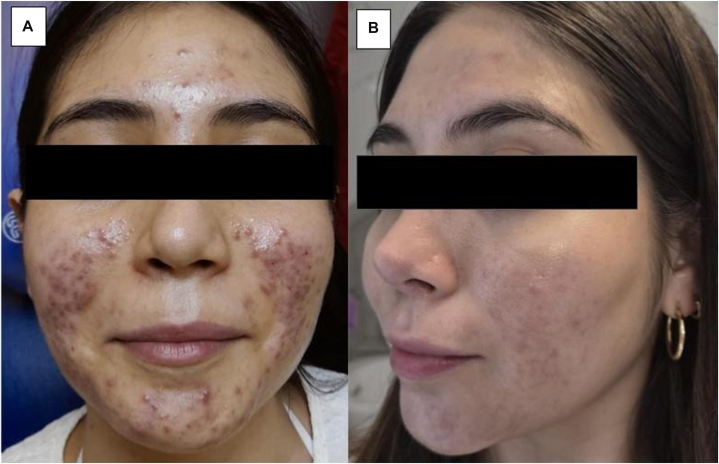
**A,** Cystic and pustular lesions after the use of 1% topical clascoterone. **B,** Resolution of lesions after discontinuation of clascoterone and initiation of benzoyl peroxide and adapalene.

One month after discontinuing oral contraconceptives, she experienced mild acne on the cheeks, with few inflammatory lesions. Topical clascoterone 1% cream was prescribed once daily at night. After 3 weeks, she presented with a severe flare consisting of painful, pruritic, cystic, and pustular lesions, accompanied by facial edema (Fig 1, *A*). Physical examination revealed marked inflammation, induration, and multiple papules and nodules on the forehead and cheeks, predominantly in the perioral area, with inflammatory plaques.

A diagnosis of severe acne induced by clascoterone was made. The drug was discontinued, and treatment was initiated with benzoyl peroxide cleansing bar and adapalene 0.1%. Progressive clinical improvement was observed during follow-up (Fig 1, *B*).

## Discussion

Acne is the eighth most prevalent disease worldwide, mainly affecting adolescents and young adults, and significantly impairs quality of life.^2^ Sebaceous gland activity is central to its pathogenesis, as androgen stimulation induces gland enlargement, increased sebum production, and local inflammatory responses.^1^^,^^6^

Clascoterone, or cortexolone 17α-propionate, is a topical steroidal androgen receptor antagonist acting on sebaceous glands and hair follicles. By competitively inhibiting dihydrotestosterone, it prevents androgen-mediated transcription of genes involved in lipid synthesis and cytokine production, thereby reducing sebum output and inflammation.^5^^,^^6^ Clinical trials have demonstrated its efficacy and favorable safety profile.^5^^,^^7^

Nevertheless, paradoxical acne exacerbations have been reported, including moderate to severe nodulocystic and conglobate forms.^7^ The underlying mechanism remains unclear. Proposed hypotheses include transient dysregulation of androgen receptor signaling before complete receptor blockade, individual susceptibility influenced by hormonal fluctuations, and local inflammatory or irritant effects of the formulation.^1^^,^^3^ In this case, recent withdrawal of oral contraceptives may have contributed to hormonal instability and increased cutaneous androgen sensitivity, acting as a predisposing factor.

Causality assessment was performed using the Naranjo Adverse Drug Reaction Probability Scale, yielding a score of 7, consistent with a *probable* adverse drug reaction. This score was based on the clear temporal relationship between clascoterone initiation and symptom onset, objective clinical documentation, absence of alternative explanations, and significant improvement after drug discontinuation. According to the World Health Organization–Uppsala Monitoring Centre causality categories, this case was classified as *probable/likely*, given the reasonable time relationship, positive dechallenge, and lack of a more plausible alternative cause.^8^

Differential diagnoses were considered. Acneiform drug eruptions typically present as monomorphic papulopustular lesions without nodules or cysts, making this diagnosis less likely. Allergic or irritant contact dermatitis was also considered; however, the presence of comedones and deep inflammatory nodules and cysts supported a true acne flare. The potential role of cortexolone, the active metabolite of clascoterone, cannot be excluded, as altered local metabolism may contribute to paradoxical inflammatory responses in susceptible individuals.

This highlights the need for clinicians to remain alert to atypical reactions despite the overall favorable safety profile of clascoterone. Compared with systemic antiandrogens such as spironolactone, clascoterone was expected to provide similar benefits without systemic adverse effects.^1^^,^^7^ However, this case emphasizes that although rare, severe paradoxical reactions may occur and warrant immediate discontinuation. Further studies are needed to clarify predisposing factors and guide management strategies in such scenarios.

## Conclusion

Clascoterone represents an important therapeutic advance in acne management, offering topical androgen blockade with minimal systemic absorption. However, this case demonstrates that it can induce severe acne, an underreported but clinically significant adverse event. Additional research is required to elucidate the mechanisms involved and to determine recommendations for prevention and treatment of this reaction.

## Conflicts of interest

None disclosed.
